# Relative tuning of holistic face processing towards the fovea

**DOI:** 10.1016/j.visres.2022.108049

**Published:** 2022-04-20

**Authors:** Teresa Canas-Bajo, David Whitney

**Affiliations:** a Vision Science Graduate Group, University of California, Berkeley, Berkeley, CA, USA; b Department of Psychology, University of California, Berkeley, Berkeley, CA, USA

**Keywords:** Holistic perception, Face perception, Mooney faces, Central bias, Foveal tuning

## Abstract

Humans quickly detect and gaze at faces in the world, which reflects their importance in cognition and may lead to tuning of face recognition toward the central visual field. Although sometimes reported, foveal selectivity in face processing is debated: brain imaging studies have found evidence for a central field bias specific to faces, but behavioral studies have found little foveal selectivity in face recognition. These conflicting results are difficult to reconcile, but they could arise from stimulus-specific differences. Recent studies, for example, suggest that individual faces vary in the degree to which they require holistic processing. Holistic processing is the perception of faces as a whole rather than as a set of separate features. We hypothesized that the dissociation between behavioral and neuroimaging studies arises because of this stimulus-specific dependence on holistic processing. Specifically, the central bias found in neuroimaging studies may be specific to holistic processing. Here, we tested whether the eccentricity-dependence of face perception is determined by the degree to which faces require holistic processing. We first measured the holistic-ness of individual Mooney faces (two-tone shadow images readily perceived as faces). In a group of independent observers, we then used a gender discrimination task to measured recognition of these Mooney faces as a function of their eccentricity. Face gender was recognized across the visual field, even at substantial eccentricities, replicating prior work. Importantly, however, holistic face gender recognition was relatively tuned—slightly, but reliably stronger in the central visual field. Our results may reconcile the debate on the eccentricity-dependance of face perception and reveal a spatial inhomogeneity specifically in the holistic representations of faces.

## 1. Introduction

Faces play a central role in our everyday life. Face perception is at the center of most social interactions, from allowing us to recognize our friends among a group of strangers to facilitating communication through non-verbal language and emotion recognition (Jack & Schyns, 2015). Humans tend to quickly direct their attention and gaze to the faces in a scene (Bindemann, Burton, Hooge, Jenkins & de Haan, 2005; Cerf, Frady & Koch, 2009; Cerf, Harel, Einhauser & Koch, 2008; Costela & Woods, 2019; Coutrot & Guyader, 2014; Foulsham, Cheng, Tracy, Henrich & Kingstone, 2010; Jack & Schyns, 2015; Marat, Rahman, Pellerin, Guyader & Houzet. 2013; Ro, Russell & Lavie, 2001; Theeuwes & Van der Stigchel, 2006) and we prefer faces to other objects from a very young age (Umiltà, Simion & Valenza, 1996; Farzin, Rivera & Whitney, 2009; Frank, Vul & Johnson, 2009). Indeed, we are highly trained with faces, as we typically see and recognize many faces every day starting in infancy. Face perception therefore enjoys a unique and privileged role in cognition.

One of the main advantages faces have over other objects is that we process them holistically – that is, we process faces as a whole instead of (or in addition to) processing the segmentable parts that make the face (eyes, nose, mouth, etc; Farah, Wilson, Drain & Tanaka, 1998; Sergent, 1984). Holistic processing develops early in humans and reaches adult-like levels in childhood (Crookes & McKone, 2009). Holistic processing has been traditionally operationalized by the magnitude of the inversion effect, the enhanced recognizability of upright faces relative to inverted ones. While upright faces are processed holistically, inverted faces are perceived via part-based processes (Yin, 1969). This gives upright faces an advantage over inverted faces—they are processed faster and more accurately (Maurer, Le Grand & Mondloch, 2002; Rossion, 2008, 2009).

Because of the critical role they play in our lives, faces are quickly identified in a scene and are often processed at the fovea. The resulting life-long experiences we have foveating faces, and the perceptual learning that accompanies this (Bi, Chen, Weng, He & Fang, 2010; Gold, Bennett & Sekuler, 1999), might therefore produce specialized mechanisms that are uniquely found in the central visual field. That is, face recognition may be retinotopically tuned to the fovea. Unfortunately, previous work has yielded mixed results on this question. Behavioral and psychophysical results have found little foveal selectivity in face recognition, whereas neuroimaging studies have provided evidence for a central-field bias in face representations.

On the one hand, previous behavioral work has shown that face recognition is relatively unaffected in the periphery up to 16° of eccentricity (McKone, 2004), at least when there is little clutter or crowding (Louie, Bressler & Whitney, 2007). Extrafoveal faces can be detected, recognized, selected, and saccades can be made to their peripheral locations faster and more accurately than to other objects (Boucart et al., 2016; Crouzet, Kirchner & Thorpe, 2010; Hershler, Golan, Bentin & Hochstein, 2010; Wolfe & Whitney, 2014). In line with these findings, holistic processing of faces also happens across the visual field (Kovács et al., 2017; Kreichman, Bonneh & Gilaie-Dotan, 2020; McKone, 2004). To investigate the extent to which faces are processed holistically in the periphery, McKone (2004) compared recognition of a Mooney face and a single nose in five visual field locations. McKone (2004) found that holistic processing happened at all tested eccentricities (up to 21 degrees in left and right visual fields) only for the whole faces. Processing of a single face feature (the nose) yielded little to no holistic processing at any eccentricity. Similar conclusions have been reached by others (Kovács et al., 2017; Kreichman et al., 2020).

On the other hand, neuroimaging work has revealed a central-field bias in face representations that does not exist as strongly for other objects (Baldassano, Iordan, Beck & Fei-Fei, 2012; Hasson, Levy, Behrmann, Hendler & Malach, 2002; Kanwisher, 2001; Kamps, Hendrix, Brennan & Dilks, 2020; Levy, Hasson, Avidan, Hendler & Malach, 2001; Sayres & Grill-Spector, 2008; Silson, Groen, Kravitz & Baker, 2016). For example, Schwarzlose and colleagues found that there was stronger BOLD response for foveally presented faces (Schwarzlose, Swisher, Dang & Kanwisher, 2008), and Nichols and colleagues found that face decoding was better in the central compared to peripheral positions (Nichols, Betts & Wilson, 2010). Similarly, population receptive fields for face stimuli in several occipito-temporal areas are biased toward central and lower visual field locations (Gomez, Natu, Jeska, Barnett & Grill-Spector, 2018; Silson et al., 2016; Kay, Weiner & Grill-Spector, 2015; Yue, Cassidy, Devaney, Holt & Tootell, 2011). Some of the foveal bias in face representations might arise from factors like cortical magnification and receptive field scaling, or to confounds including SNR differences within and across brain regions. However, face and object selective regions are highly selective to visual location, even in the periphery (e.g., multivoxel pattern analysis revealed retinal selectivity of 0.14E in FFA, which indicates that a face shifted by 14% of its eccentricity was reliably discriminated, even for objects at 9 degrees eccentricity; Fischer & Whitney, 2011). In addition to this, other confounds like variations in SNR are not thought to be entirely responsible for the visual field biases in face representations (Silson et al, 2016; Poltoratski, Kay & Grill-Spector, 2019). There is therefore sufficient and converging evidence to suggest that face representations are stronger or biased (or perhaps remapped; Williams et al., 2008; Edwards, VanRullen & Cavanagh, 2018; Wolfe & Whitney, 2014) toward the fovea (Hasson et al., 2002; Kay et al., 2015; Yue et al., 2011; Silson et al., 2016; Sayres & Grill-Spector, 2008). Such biases in face representations may speak to the importance of perceptual learning: we tend to foveate faces in natural scenes, as they have crucial information for social communication and interaction (Costela & Woods, 2019; Jack & Schyns, 2015; Coutrot & Guyader, 2014). However, the neuroimaging findings beg the question of whether there is some behavioral consequence of the central visual field bias in face representations, and why previous psychophysical results have not clearly found it.

**Fig. 1.**
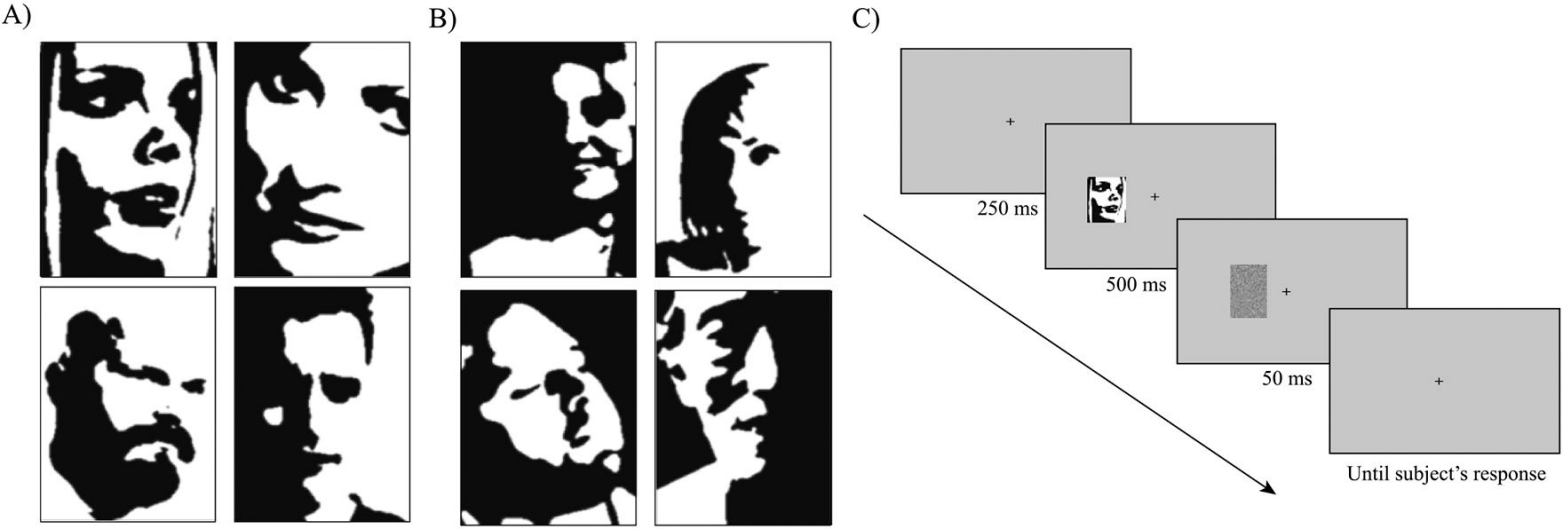
A) Four sample two-tone faces in the low-holistic group. B) Four sample Mooney faces in the high-holistic group. The two groups were formed based on the results of pilot study B with independent subjects (details in the Methods section). All stimuli were extracted from Schwiedrzik et al. (2018). C) Illustration of experiment procedure. Faces not drawn to scale.

Here, we propose addressing these mixed results by testing for any degree of foveal tuning of holistic face perception. In other words, we hypothesize that there may be a relative preference or relative strength of holistic processing of faces at the fovea compared to the periphery. To isolate holistic processing, we used a more stringent stimulus-specific operational definition of holistic perception than is typically used. To test for the eccentricity-dependence of face gender recognition, we controlled for the degree of holistic processing required by each specific stimulus. Previous work shows that some faces tap into holistic perception more readily than others (Canas-Bajo & Whitney, 2020), so we isolated the most holistically processed faces. In the following experiment, we used Mooney faces (Fig. 1A), which are two-tone images that are readily perceived as faces even though they lack segmentable face features (Mooney, 1957). Mooney faces are a special set of two-tone images because they maintain the minimal stable structure of faces (Ke, Yu & Whitney, 2017). Because these faces don’t have easily segmented face features, they have to be processed as a whole to be perceived as faces, which makes them ideal stimuli to test holistic perception (Andrews & Schluppeck, 2004; Bona, Cattaneo & Silvanto, 2016; Cavanagh, 1991; Farzin et al., 2009; Latinus & Taylor, 2005; McKone, 2004; Moore & Cavanagh, 1998). However, previous work shows that there are some two-tone images that do retain some featural information and can be processed by parts and/or holistically in parallel (Canas-Bajo & Whitney, 2020). These two-tone faces would therefore not be ideal Mooney faces, per se, since they do not isolate holistic processing. In contrast, purely holistic Mooney faces can only be processed holistically: one must first recognize the whole image as a face before any particular feature or fragment can be identified as, for example, an eye, nose, or mouth (Cavanagh, 1991). Likewise, one must also recognize the image as a face before the gender of identity of the Mooney face can be discriminated (Cavanagh, 1991; Farzin et al., 2009). By testing the extent of holistic processing across the visual field for relatively holistic faces, we aim to investigate 1) whether there is indeed a central-field bias for face processing and 2) whether it exists exclusively for holistic faces. To foreshadow our results, faces are readily recognized throughout the visual field, and, interestingly, we found a slight foveal tuning for purely holistic Mooney face gender recognition, such that holistic Mooney face perception was relatively stronger near the fovea.

## 2. Method

### 2.1. Participants

Twenty-four participants took part in the experiment. All subjects were undergraduate students at the University of California, Berkeley and provided written consent before the start of the experiment. The UC Berkeley Institutional Review Board granted ethics approval for the study.

### 2.2. Material

The stimuli consisted of forty images of Mooney faces extracted from Schwiedrzik, Melloni and Schurger (2018). To select these faces, we ran two pilot experiments (pilot experiment A and B) with independent observers: one that tested the gender of the faces and another one that measured the extent to which the faces tapped into holistic processing. The presentation of the stimuli and the data collection of both pilot experiments was done online and was controlled using Qualtrics (https://www.qualtrics.com). All stimuli presented in the pilot experiments were of size 160 × 230 pixels but note that participants used different monitors and distance to monitor could not be controlled.

In pilot experiment A, our goal was to determine the ground truth gender of each face. Because we did not have access to the original grayscale face images from which the Mooney faces were generated, and because gender appearance is subjective in any case (Freeman, Rule, Adams & Ambady, 2010), we asked twenty independent observers to categorize the gender of a random sample of 274 faces from the original Schwiedrzik et al. (2018) Mooney dataset (about half of that original dataset). Each trial consisted of a Mooney face that was presented at the center of the screen until participants recorded a response. The gender of 216 faces was rated with 90% of subject agreement.

In pilot experiment B, our goal was to measure how holistic each Mooney face image was. The present study aimed to compare eccentricity-dependent performance in two groups of faces: high-holistic faces and low-holistic faces. High holistic Mooney faces are those that isolate holistic processing while low holistic two-tone faces are those that can be processed holistically and in a part-based manner. Following the procedure in Canas-Bajo & Whitney (2020), to quantify the extent to which each face tapped into holistic processing, we measured the magnitude of the inversion effect for each face in pilot experiment B with twenty new, independent observers. Here, participants were asked to categorize the gender of the 216 Mooney faces selected in pilot experiment A. Each face was presented once upright and once inverted across the experiment, and the inversion effect for each face was quantified as the difference in accuracy between the upright and inverted condition. Faces were presented foveally until participants recorded a response. Participants were allowed to move their eyes. Overall, we found the well-known inversion effect: gender recognition of upright faces was more accurate (Mean: 0.97, SEM: 0.0) than of inverted faces (Mean: 0.85, SEM: 0.01). The magnitude of the inversion effect however varied across Mooney faces, replicating Canas-Bajo & Whitney (2020). Out of the 216 faces tested, we selected the 20 faces with the weakest inversion effect (Mean: 0.0, SEM: 0.0) and the 20 faces with the strongest inversion effect (Mean: 0.41, SEM: 0.03), *t*(19) = 0.0, *p* < 0.001. From now on, we will refer to these two groups of faces as the “low-holistic” group (Fig. 1A) and “high-holistic” group (Fig. 1B), respectively.

In the main study, stimuli were presented on a CRT monitor at 100 Hz refresh rate, with 1024 × 768 pixels resolution and a horizontal screen size of 40.5 cm. The monitor was placed 60 cm from a chin rest that stabilized the participant’s head. At this distance, all the face stimuli shown during the experiment subtended 6 degrees visual angle. The presentation of the stimuli was controlled using MATLAB R2016b with Psychophysics Toolbox 3 (Brainard, 1997; Kleiner, Brainard, & Pelli, 2007).

### 2.3. Design

Each subject completed a total of 1300 trials: (2 orientation conditions (Upright vs. Inverted) × 2 face conditions (High-holistic vs. low-holistic face group) × 13 locations (2°, 4°, 6°, 8°, 10° and 12° in both the right and left visual field, as well as the fovea) × 25 repetitions. Each orientation-face-location condition was repeated 25 times.

### 2.4. Procedure

Participants performed a gender-discrimination task in which they had to determine the gender of an upright or inverted Mooney face at different visual field locations. We chose a gender discrimination task because it was ideal for our goals and stimuli: First, we needed unfamiliar faces and yet Mooney faces, like shadow-defined shapes, are not identifiable unless they are very familiar identities (Moore & Cavanagh, 1998). Second, a face detection or face/non-face discrimination task would have required relying on “scrambled” face lures—tasks and stimuli that are subject to, and can introduce, bias. Lastly, the goals of our study necessitated a task that required holistic processing, and Mooney gender discrimination requires holistic processing (Sergent & Signoret, 1992; Steeves et al., 2006; Yokoyama, Noguchi, Tachibana, Mukaida, & Kita, 2014; Zhao & Hayward, 2010). Although previous literature using grayscale faces shows that featural information may be used to determine the gender of a face (Brown & Perrett, 1993; Schyns, Bonnar, & Gosselin, 2002; Nestor & Tarr, 2008; Hu, Hu, Xu, & Qin, 2013; Yamaguchi, Hirukawa, & Kanazawa, 2013), others have shown the importance of holistic processing in face gender discrimination (Yokoyama et al., 2014; Zhao & Hayward, 2010). More importantly, because we used Mooney faces, holistic processing plays a key role in our face gender discrimination task (Sergent & Signoret, 1992; Steeves et al., 2006; Yokoyama et al., 2014; Zhao & Hayward, 2010).

A trial sequence started with a 250 ms (ms) fixation cross. Next, a face was shown for 500 ms at one of the thirteen possible locations. Participants were instructed to ignore the orientation of the face. Immediately after the face disappeared, a noise mask was shown for 50 ms. A grey blank screen was displayed until a response was given (self-paced). The trial ended with a 500 ms inter-trial-interval. Subjects were asked to fixate at the center of the screen throughout the trial and avoid any eye movements. Fig. 1C illustrates a summary of the design and procedure of the experiment.

### 2.5. Data analysis

Here, we operationalized the extent of holistic processing by the magnitude of the inversion effect (Yin, 1969). The inversion effect for each location and face group (high vs. low-holistic) was calculated by subtracting the mean accuracy in the inverted from the upright conditions. We performed a three-way repeated measures ANOVAs with Orientation (upright vs. inverted), Face group (high vs. low-holistic group) and Eccentricity (13 locations tested) as factors and accuracy as the dependent variable. We also performed a two-way repeated measures ANOVA with Face group and Eccentricity as factors and magnitude of the inversion effect as the dependent variable. To test the significance of some effects, we calculated the null distributions using permuting and bootstrapping procedures with 10,000 iterations (Efron & Tibshirani, 1994). All reported pair-comparisons p values were corrected using Benjamini–Hochberg (BH) procedure (Benjamini & Hochberg, 1995).

## 3. Results

The main goal of this experiment was to investigate the eccentricity-dependence of face gender recognition and whether it depends on the “holisticness” of each face. Here, we operationalized holistic processing as the magnitude of the inversion effect. The inversion effect is the accuracy in the upright condition minus the accuracy in the inverted condition. Thus, we first calculated the average in the upright and inverted conditions, separately, for each face group at each location. We applied a three-way (Orientation × Face group × Eccentricity) repeated measures ANOVA for upright and inverted accuracies.

**Fig. 2.**
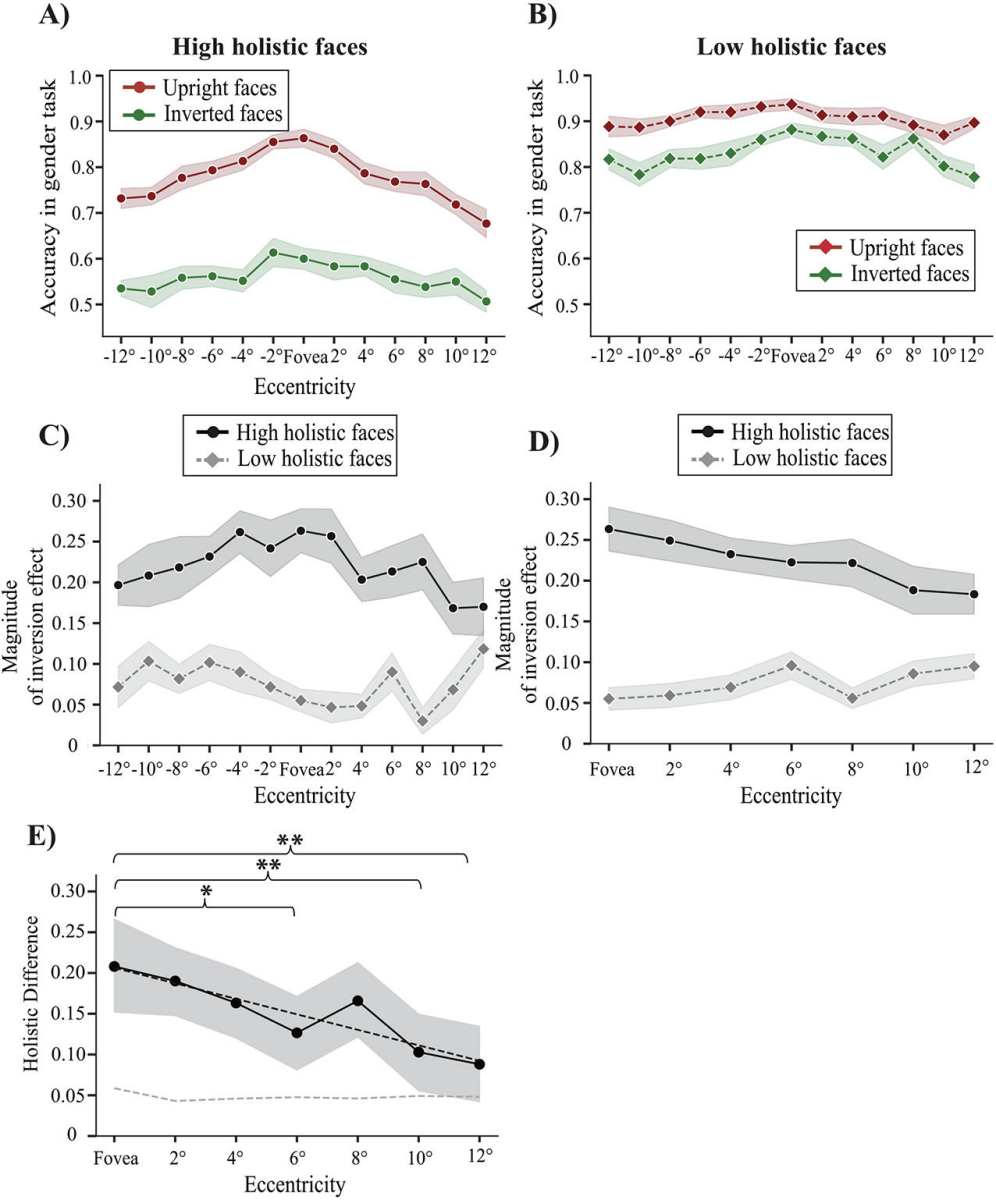
Results of Experiment 1. A) Gender recognition of high-holistic faces that were upright (red) or inverted (green). Eccentricity is shown on the abscissa. B) Gender recognition of low-holistic faces that were upright (red) or inverted (green) as a function of eccentricity. C) Magnitude of the inversion effect for high- (dark grey) and low- (light grey) holistic faces. The inversion effect is operationalized as the difference between accuracy for upright minus inverted faces at each eccentricity. D) Magnitude of the inversion effect as a function of unsigned eccentricity for high- (dark grey) and low- (light grey) holistic faces. The shaded region represents the standard error of the mean between subjects in panels A-C. E) Difference between inversion effect (blue) for high versus low holistic faces. Stars represent the significance of the pair comparisons of holistic difference between eccentricities, one star represents p < 0.05, two stars represents p < 0.01, BH-corrected. The shaded grey region represents the bootstrapped 95% confidence intervals. The dashed-dotted black line represents the linear regression fit to the data. The dashed grey line represents the 97.5% upper bound of the permuted null difference.

Both low- and high-holistic upright faces were recognized above chance in the periphery (maximum tested eccentricity was 12 degrees; Low holistic: mean = 0.89, SEM = 0.01; High holistic: mean = 0.70, SEM = 0.02; Fig. 2A-B). This result replicates and extends prior work (e.g., McKone, 2004). For both low- and high-holistic groups, the accuracies for the upright condition were higher than for the inverted condition, *t*(23) = 12.87, *p* < 0.001, *η*^2^ = 0.24 and *t*(23) = 25.06, *p* < 0.001, *η*^2^ = 0.69, replicating the well-studied inversion effect. Unsurprisingly, the difference between upright and inverted faces at each location was larger in the high-holistic group than in the low-holistic group, revealed by a significant interaction between Orientation and Face group, *F*_2,46_ = 174.50, *p* < 0.001, *η*^2^ = 0.08. This was expected, because the division between high- and low-holistic face stimuli was based on independent observers in pilot testing (see Methods). In fact, gender recognition of high-holistic inverted faces remained close to chance across all eccentricities (Mean = 0.56, SEM = 0.02; Fig. 2A); this conforms to the definition of a holistic face being one that is easily recognizable when upright but not when inverted. In contrast, low-holistic faces were recognized well in the periphery with almost no decrement up to 12 degrees in both upright and inverted conditions (Upright: Mean = 0.89, SEM = 0.02; Inverted: Mean = 0.81, SEM = 0.02; Fig. 2B). Importantly, we found a significant interaction between face group and eccentricity for the upright condition (*F*_12,276_ = 3.48, *p* < 0.001, *η*^2^ = 0.03). Accuracy for upright high-holistic faces was 0.85 (SEM = 0.02) at the fovea, but then performance decreased as faces appeared further in the periphery (Fig. 2A, red curve). In contrast, gender recognition of upright low-holistic faces remained relatively unaffected in the periphery compared to the fovea (Upright: Mean = 0.89, SEM = 0.02; Fig. 2B, red curve). This result suggests a relative foveal tuning in holistic processing of upright faces: a slightly stronger preference for purely holistic faces at the fovea, compared to the periphery.

To clarify the interaction between eccentricity and degree of holistic processing, we quantified the magnitude of the inversion effect for each face group at each eccentricity. A two-way repeated measures ANOVA on the magnitude of the inversion effect revealed a significant effect of Face group, *F*_1,23_ = 70.32, *p* < 0.001, *η*^2^ = 0.26. Unsurprisingly, we found that high-holistic faces had an overall stronger inversion effect than low-holistic faces, *t*(24) = 7.74, *p* < 0.001, *η*^2^ = 0.53. As discussed above, the a-priori hypothesis is that there may be an effect of eccentricity on face gender recognition. There is no expectation of a left versus right visual field difference, and, indeed, there is no significant difference between left and right visual fields for any condition (all *ps* > 0.05). Because our primary interest is eccentricity, we collapsed across isoeccentric locations in the left and right visual field (Fig. 2D). Low-holistic faces remained relatively unaffected up to 12 degrees of eccentricity while high-holistic faces showed a relatively stronger inversion effect at the fovea that decreased gradually with eccentricity. There was a significant interaction between Face group and Eccentricity (seven eccentricities total), *F*_6,138_ = 2.97, *p* < 0.01, *η*^2^ = 0.03.

To help visualize this interaction, we calculated the holistic difference at each eccentricity (Fig. 2E). That is, the difference in magnitude of the inversion effect between high and low-holistic faces at each eccentricity. The holistic difference revealed that there was a larger difference between high and low-holistic face groups at the fovea than other eccentricities (Fig. 2E). To further test the significance of this interaction, we calculated the null distribution for the interaction by shuffling the face group label for each location in a nonparametric permutation procedure. We found that the difference in inversion effect between high and low-holistic face group was significant at all locations up to 10 degrees (*p* < 0.05 per permutation analysis) and importantly, was largest at the fovea (compared to 6, 10 and 12 degrees, *p* < 0.05 BH-corrected; Fig. 2E). A linear regression fit to the data further confirmed this result (n = 168, *R*^2^ = 0.07, *p* < 0.01). This result further suggested that there is some degree of foveal tuning of face gender recognition that is specific to holistic faces.

**Fig. 3.**
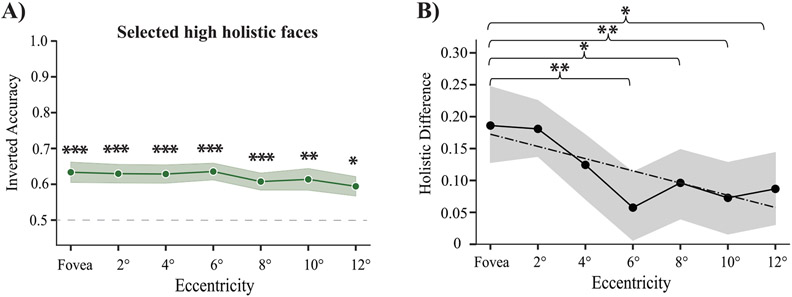
Control analysis. A) Inverted faces that are recognized greater than 50% of the time were selected for subsequent analyses. The shaded region represents the standard error of the mean between subjects. Asterisks indicate statistical significance (one star represents p < 0.05, two stars represents p < 0.01, three stars represent p < 0.001, BH-corrected). B) Difference between inversion effect for high versus low holistic faces that were selected. The ordinate shows the holistic difference score (as in Fig. 2E). Stars represent the significance of the paired comparisons of holistic difference between the fovea and each eccentricity, one star represents p < 0.05, two stars represents p < 0.01, BH-corrected. The dashed-dotted black line represents the linear regression fit to the data. The shaded grey region represents the bootstrapped 95% confidence intervals.

In addition, we tested whether the foveal tuning found in high holistic faces was due to floor performance in high holistic inverted faces across the visual field. The goal of this analysis was to rule out the possibility that floor or ceiling performance alone may cause the relatively stronger foveal tuning in high holistic faces. To this end, we identified inverted faces with an accuracy above 50% (average accuracy ~ 60% across all eccentricities; all *ps* < 0.05; Fig. 3A). These selected high and low holistic faces are not at floor. Next, we calculated the inversion effect for the low and high holistic groups separately including only those faces. Lastly, we tested whether this group of above-floor faces showed similar foveal tuning specific to high holistic faces by examining the difference in magnitude of the inversion effect between high and low-holistic faces at each eccentricity. If the at-chance inverted accuracy in high holistic faces drives the pattern of foveal tuning, then we should see that this pattern does not persist when we limit the analysis to faces that have significantly above-chance accuracy. The holistic difference revealed that high holistic faces were recognized more readily at the fovea than at any other eccentricity, and this persisted when we restricted the analysis to faces with above-chance inverted accuracy (Fig. 3B). There was a significant difference for fovea versus 6-, 8-, 10-, and 12-degree eccentric faces (all *ps* < 0.05, BH-corrected). A linear regression fit to the data confirm this tuning (n = 168, *R*^2^ = 0.06, *p* < 0.01). In sum, our results show that there is a relative foveal tuning in holistic face processing of upright faces that is not explained by a floor performance in inverted faces.

## 4. Discussion

In the present study, our goal was to reconcile an existing debate on the eccentricity-dependence of face perception. Behavioral studies have shown that faces are relatively unaffected by the constraints of peripheral vision and that holistic processing happens across the visual field (Kovács et al., 2017; Kreichman et al., 2020; McKone, 2004) whereas brain imaging studies have provided evidence for a central-field bias in faces that does not exist for other objects (Amedi, Malach, Hendler, Peled & Zohary, 2001; Gomez et al., 2018; Grill-Spector & Weiner, 2014; Hasson et al., 2002; Kanwisher, 2001; Kay et al., 2015; Levy et al., 2001; Malach, Levy & Hasson, 2002; Silson et al., 2016; Yue, et al., 2011). Here, we aimed to resolve this controversy by investigating to what extent the eccentricity-dependence of face perception is determined by the degree to which holistic processing is required to process each face. In order to isolate holistic processing from faces that can be processed by parts, we categorized two-tone faces into high-holistic Mooney faces (that required holistic processing) and low-holistic two-tone faces (that relied less on holistic processing and can be processed by parts and/or holistically), following the method in Canas-Bajo & Whitney, 2020. Replicating previous work in the field, we found that upright faces, holistic or not, are well recognized at 12 degrees in the periphery or beyond (Farzin et al., 2009; Kovács, et al., 2017; McKone, 2004). Also, in accordance with previous findings, our results show that holistic processing occurs for peripheral faces, even at very large eccentricities (McKone, 2004). The novel result in our study is the dissociation in the effect of eccentricity on high and low-holistic face gender recognition. We observed that holistic faces were foveally tuned, which means that holistic processing was slightly but reliably stronger at the fovea than in the periphery. Together, our results shed light on the discrepancy between psychophysical and brain imaging studies: there is indeed some degree of face processing tuned toward the fovea, but it is particularly stronger for purely holistic faces.

In natural visual search and recognition tasks, observers tend to prefer to foveate faces (e.g., Boucart, et al, 2016; Xia, Manassi, Nakayama, Zipser & Whitney, 2020; Martin, Davis, Riesenhuber, & Thorpe, 2018), and observers readily recognize peripheral faces (McKone, 2004; Farzin et al., 2009) and make accurate saccades to them (Farzin, Rivera & Whitney, 2010; Crouzet et al., 2010; Martin et al, 2018; Wolfe & Whitney, 2015). We foveate faces at least in part because face recognition improves with access to fine detail (Goffaux, Hault, Michel, Vuong & Rossion, 2005; Kwon & Legge, 2011) and for other reasons like lip reading (Lansing & McConkie, 2003; Schwartz, Berthommier, & Savariaux, 2004). As a consequence, humans learn to recognize faces by foveating them, and we generally have more experience foveating faces. This could potentially lead to stronger or more numerous holistic templates in the central visual field compared to the peripheral visual field.

The foveal tuning for holistic processing found here is consistent with the extensive brain imaging literature that shows a central visual-field bias for faces which does not exist for other objects (Amedi et al., 2001; Baldassano et al., 2012; Grill-Spector & Weiner, 2014; Hasson et al., 2002; Kamps, Hendrix, Brennan & Dilks, 2020; Kanwisher, 2001; Levy et al., 2001; Malach et al., 2002; Sayres & Grill-Spector, 2008; Silson et al., 2016). That is, face representations are more affected by eccentricity than other objects (Kreichman et al., 2020; Schwarzlose et al., 2008). Our results also support this retinotopic organization of face processing (Hasson et al., 2002; Levy et al., 2001), but they also extend and clarify it, revealing an explanation for the reported foveal bias in neuroimaging results. In particular, our results help explain how commonly used neuroimaging methods reveal and reinforce the apparent foveal bias in face representations. For example, neuroimaging studies using univariate and multivariate approaches commonly contrast or classify the differences between object and face responses (e.g., using multivariate pattern analysis to classify faces versus objects). Comparing (implicitly or explicitly) responses to upright faces relative to either objects or inverted faces could create a bias that more heavily weighs holistic processes. Our psychophysical results show that these holistic processes are relatively tuned toward the central visual field. This naturally leads neuroimaging studies to estimate (and potentially overestimate) the foveal tuning of face processing. Our results suggest that the central-field bias does, in fact, reflect a spatial inhomogeneity in face processing—a modest but consistent strength in the holistic representations of faces.

Our results help reconcile the debate on the eccentricity-dependence of face perception, and they make a prediction: faces that are processed by parts (fractured faces, or two-tone faces that maintain featural information, for example) will not result in the kind of foveal bias that the neuroimaging literature has reported. Additionally, those fractured faces will not have such a strong foveal bias when measured psycho-physically either. In contrast, faces that require holistic processing, such as hard Mooney faces, will show stronger tuning to the fovea.

Within-face crowding has been shown to impair recognition of grayscale face features in the periphery (Kalpadakis-Smith, Goffaux & Greenwood, 2018; Martelli, Majaj & Pelli, 2005; Liu, Montaser-Kouhsari & Xu, 2014). Our results show that Mooney faces (especially relatively low-holistic Mooney faces) are well-recognized across the visual field. From this and previous work on peripheral recognition of Mooney faces (Farzin et al., 2009; Farzin et al., 2010; Goold & Meng, 2016), it may be that Mooney faces do not suffer within-face crowding to the same extent that grayscale faces do. We propose two reasons to explain the weaker within-face crowding in our experiment. First, the task tested here required discerning the gender of two-tone faces, which is a demanding task that involves recognizing global and configural cues and relies on holistic processing, especially for those high holistic Mooney faces that isolate holistic processing (Yokoyama et al., 2014; Zhao & Hayward, 2010). Within-face crowding has only been shown for single feature discrimination tasks, like recognizing the shape of the mouth or eyes (Martelli et al., 2005; Liu, Montaser-Kouhsari & Xu, 2014). For true holistic tasks (identity and gender recognition of Mooney faces, for example), crowding also happens between whole faces (Farzin et al., 2009; Louie et al., 2007). Second, the weaker within-face crowding in Mooney faces may support two parallel ways to recognize the gender of a face. Even if there were crowding between the features of the face (thus impairing recognition of a particular eye or nose), there could still be sufficient holistic information about the structure of the face or its identity that could give away information about the gender of the face (e.g., Fischer & Whitney, 2011).

Holistic and part-based perception have been defined as two independent systems that can operate simultaneously and in parallel (McKone, 2004; Moscovitch, Winocur & Behrmann, 1997). The redundancy in face processing pathways has several advantages. These include 1) a degree of resilience to brain damage (Boutsen & Humphreys, 2002; Moscovitch et al., 1997; Rivest, Moscovitch & Black, 2009); 2) a plausible way to release crowding (Manassi & Whitney, 2018); and 3) increased resources devoted to face recognition in the central visual field while still maintaining robust face detection mechanisms throughout the periphery (Boucart et al., 2016). The redundancy in face recognition processes also results in complex interactions in face recognition across the visual field. A face that isolates holistic processing, such as our high holistic Mooney faces, can only be processed by the holistic system, and its recognition is therefore somewhat easier in the central visual field. A low-holistic face might benefit from both systems (holistic and part-based processes) and thus can be recognized more easily across the visual field, which would explain the overall better gender recognition of low holistic faces in our data.

A limitation of this study is that it does not address task-dependent effects on the tuning of faces across the visual field. It is possible that the dissociation of results found in the neuroimaging and behavioral literature arises in part from a difference in the tasks employed. In our study, we used a gender discrimination task because it allowed us to test a large sample of faces while avoiding “scrambled” face lures that can introduce bias. Previous literature has shown that gender and identity recognition are intimately linked (Baudouin & Tiberghien, 2002; Ganel & Goshen-Gottstein, 2002; Sergent & Signoret, 1992; Zhao & Hayward, 2013). This may be especially true for Mooney faces: Sergent and Signoret (1992) found that prosopagnosic patients show impaired Mooney face identity and gender recognition but not grayscale face gender recognition. That impaired identity recognition and impaired gender recognition are coupled specifically in Mooney faces suggests that a Mooney gender task such as ours likely involves similar mechanisms. Based on the past literature, we have no reason to believe the choice of our task would be an influencing factor in our study. Nevertheless, future research should investigate the importance of the task on face processing across the visual field more directly.

It is worth noting that holistic processes may not be limited to faces (Bukach, Phillips, & Gauthier, 2010; Busey, & Vanderkolk, 2005; Wong, Palmeri & Gauthier, 2009). This is hotly debated, of course, but to the extent that holistic representations are useful in non-face object and pattern recognition, the results here might apply. This could have consequences in applied settings. For example, in medical image perception, clinicians and pathologists routinely make consequential perceptual decisions about lesions in radiographs and photographs (Waite et al., 2019). These life-and-death decisions are based on pattern and object recognition abilities, but what mechanisms underlie these has remained elusive (Kok, Van Geel, van Merriënboer, & Robben, 2017). Holistic processes might be involved; for example, recent work has suggested that there are some inversion effects in lesion recognition (Chin, Evans, Wolfe, Bowen & Tanaka, 2018; Sheridan & Reingold, 2017), and this inversion effect can be stronger in expert radiologists. This echoes face recognition inversion effects and is a striking finding. It indicates that holistic representations or pathways can be perceptually learned, and that lesion recognition depends on expertise. However, an inversion effect itself does not rule out innate mechanisms. There could be self-selection in career trajectory or selection-bias during training, as well. This might seem fanciful, but face recognition (a kind of pattern recognition) is largely genetically determined (Wilmer et al., 2010). Whether the same is true for general pattern recognition is unclear. Likewise, whether holistic-based medical image perception is something that can be learned remains an outstanding question. It is therefore important to understand how holistic processing develops, what kind of perceptual learning is required, and what degree of expertise is involved. The approach employed here and elsewhere—using two-tone Mooney images to test for holistic processes—could be useful in medical image perception domains as well.

In sum, our results suggest that there is a foveal tuning in holistic processing of Mooney faces. This finding reconciles the debate in the literature between behavioral studies that suggest relatively unimpaired face perception in the periphery and brain imaging studies that suggest a central-field bias for face representations. The foveal tuning found for faces is specifically stronger for holistic processing. The results support two face recognition pathways that can work in parallel, which, in combination, support the two complementary goals of search and scrutiny: robust face detection throughout the visual field with concurrent highly detailed and precise representations of upright faces in the central visual field.
